# Bisphenol A Exposure In Utero Disrupts Early Oogenesis in the Mouse

**DOI:** 10.1371/journal.pgen.0030005

**Published:** 2007-01-12

**Authors:** Martha Susiarjo, Terry J Hassold, Edward Freeman, Patricia A Hunt

**Affiliations:** 1 Department of Genetics, Case Western Reserve University, Cleveland, Ohio, United States of America; 2 School of Molecular Biosciences and Center for Reproductive Biology, Washington State University, Pullman, Washington, United States of America; 3 Department of Biology, St. John Fisher College, Rochester, New York, United States of America; Stowers Institute for Medical Research, United States of America

## Abstract

Estrogen plays an essential role in the growth and maturation of the mammalian oocyte, and recent studies suggest that it also influences follicle formation in the neonatal ovary. In the course of studies designed to assess the effect of the estrogenic chemical bisphenol A (BPA) on mammalian oogenesis, we uncovered an estrogenic effect at an even earlier stage of oocyte development—at the onset of meiosis in the fetal ovary. Pregnant mice were treated with low, environmentally relevant doses of BPA during mid-gestation to assess the effect of BPA on the developing ovary. Oocytes from exposed female fetuses displayed gross aberrations in meiotic prophase, including synaptic defects and increased levels of recombination. In the mature female, these aberrations were translated into an increase in aneuploid eggs and embryos. Surprisingly, we observed the same constellation of meiotic defects in fetal ovaries of mice homozygous for a targeted disruption of ERβ, one of the two known estrogen receptors. This, coupled with the finding that BPA exposure elicited no additional effects in ERβ null females, suggests that BPA exerts its effect on the early oocyte by interfering with the actions of ERβ. Together, our results show that BPA can influence early meiotic events and, importantly, indicate that the oocyte itself may be directly responsive to estrogen during early oogenesis. This raises concern that brief exposures during fetal development to substances that mimic or antagonize the effects of estrogen may adversely influence oocyte development in the exposed female fetus.

## Author Summary

The potential effects on reproduction of chemicals with hormone-like activity is a growing concern. One estrogenic chemical, bisphenol A (BPA), has received considerable attention because low-dose exposures have been reported to induce a variety of reproductive effects in rodents. In the course of studies to assess the effects of BPA on the mouse oocyte, we have uncovered a novel “grandmaternal” effect: exposure to BPA during pregnancy disturbs oocyte development in unborn female fetuses. When these fetuses reach adulthood, the perturbations are translated into an increase in chromosomally abnormal eggs and embryos. Thus, low-dose BPA exposure during pregnancy has multigenerational consequences; it increases the likelihood of chromosomally abnormal grandchildren. Our studies also provide mechanistic insight, and, surprisingly, suggest that BPA acts in the fetal ovary not by mimicking the actions of estrogen but by interfering with the function of one of the known estrogen receptors. Thus, our data suggest that estrogen plays a far earlier role in oocyte development than previously suspected and, importantly, raise the possibility that a variety of substances—both synthetic and naturally occurring—that mimic the actions of estrogen or act as estrogen antagonists may affect early oocyte development.

## Introduction

The link between exposure to synthetic chemicals that mimic the actions of endogenous hormones and risks to human health is a growing concern. As early as 1970, Herbst and Scully reported vaginal clear-cell adenocarcinoma in six 14- to 21-y-old women exposed in utero to the synthetic estrogenic drug diethylstilbestrol (DES) [1]. This rare cancer had been reported previously only in elderly women, and subsequent studies confirmed an increased incidence among daughters of women given DES during pregnancy to prevent miscarriage (reviewed in [2]). Other reproductive effects have been suggested, but definitive evidence has been obtained only in experimental animals (reviewed in [3]). The DES experience has not only heightened awareness of the possible health effects of synthetic compounds that mimic the actions of hormones, but, importantly, it demonstrates the difficulty of assessing effects in humans—even when the exposure is of known duration and dose.

Bisphenol A (BPA) was formulated around the same time as DES, but, because it was considered a less potent estrogen, it was never used clinically. We are, however, exposed to BPA daily; it is a component of polycarbonate plastics, resins lining food/beverage containers, and additives in a variety of consumer products. Over 6 billion pounds are produced worldwide annually, and several studies have reported levels of BPA in human tissues in the parts per billion range [4–6].

Short-term exposure to environmentally relevant doses of BPA has been linked to a variety of reproductive effects in laboratory rodents, including reduced sperm production, alterations in prostate development, and increased susceptibility to prostate carcinogenesis in the male [7,8] and alterations in mammary gland organization, brain development, and estrous cyclicity in the female [9–11].

Our laboratory is interested in the possible effects of BPA on the genetic quality of gametes. Low-dose BPA exposure in vivo during the final stages of oocyte growth [12] or in vitro during the resumption and completion of the first meiotic division [13] disrupts meiotic chromosome behavior, resulting in the production of chromosomally abnormal eggs. Mammalian oogenesis, however, is a complex process that is initiated during fetal development but not completed until after fertilization. Hence, defining critical exposure periods requires assessment of the effects of fetal, neonatal, and adult exposures. We summarize here the results of meiotic studies of females exposed to low (20 μg/kg/day), environmentally relevant doses of BPA during a 1-wk fetal exposure. Our studies reveal a unique set of meiotic defects in BPA-exposed females and demonstrate that a knockout of one of the two known estrogen receptors phenocopies fetal BPA exposure. Together, these findings provide the first known demonstration that early meiotic events in the fetal ovary are responsive to estrogen.

## Results

### Aberrant Meiotic Prophase in BPA-Exposed Females

During fetal development, germ cells in both sexes undergo massive mitotic proliferation. Subsequently, germ cells in the testis enter mitotic arrest and remain quiescent until after birth, while those in the ovary initiate meiosis. The prophase events of female meiosis (i.e., pairing, synapsis, and recombination between homologous chromosomes) occur during fetal development. By the time of birth, oocytes have entered a protracted period of meiotic arrest, where they remain until just prior to ovulation. Resumption and completion of the first meiotic division occurs only after an extensive period of follicle growth in the adult ovary, and this occurs weeks, years, or even decades (depending on the species) after the initiation of meiotic arrest.

To assess the effect of BPA exposure during the fetal stages of oogenesis, we implanted time-release BPA pellets (designed to leach a low, environmentally relevant dose of 20 μg/kg body weight/day, as used in our previous studies of BPA exposure in young adult females [12]) or placebo pellets in pregnant C57BL/6 females at gestation day 11.5. Because the first cohort of cells initiates meiosis at 13.5 d of gestation, this ensured low-level, continuous exposure of all oocytes during meiotic prophase. To test the efficacy of this delivery system, we used an oral dosing strategy [12] on a subgroup of pregnant females. The results of the two exposure paradigms were indistinguishable (unpublished data), and all data presented here were obtained using the pellet delivery method. For the prophase studies detailed below, five replicate experiments were conducted. In each, fetuses from one BPA and one placebo-treated pregnant female were examined, with the number of females per litter ranging from one to three. Significant variation among experiments was not observed, and the data presented below represent the pooled data.

To analyze meiotic prophase, ovaries were isolated from female fetuses at 18.5 d of gestation and meiotic preparations made as described previously [14]. We analyzed the relative proportion of cells in the prepachytene, pachytene, and diplotene stages and found similar profiles in ovaries from placebo and BPA-exposed females, suggesting that the rate of progression through prophase was not affected by BPA exposure (unpublished data).

**Figure 1:**
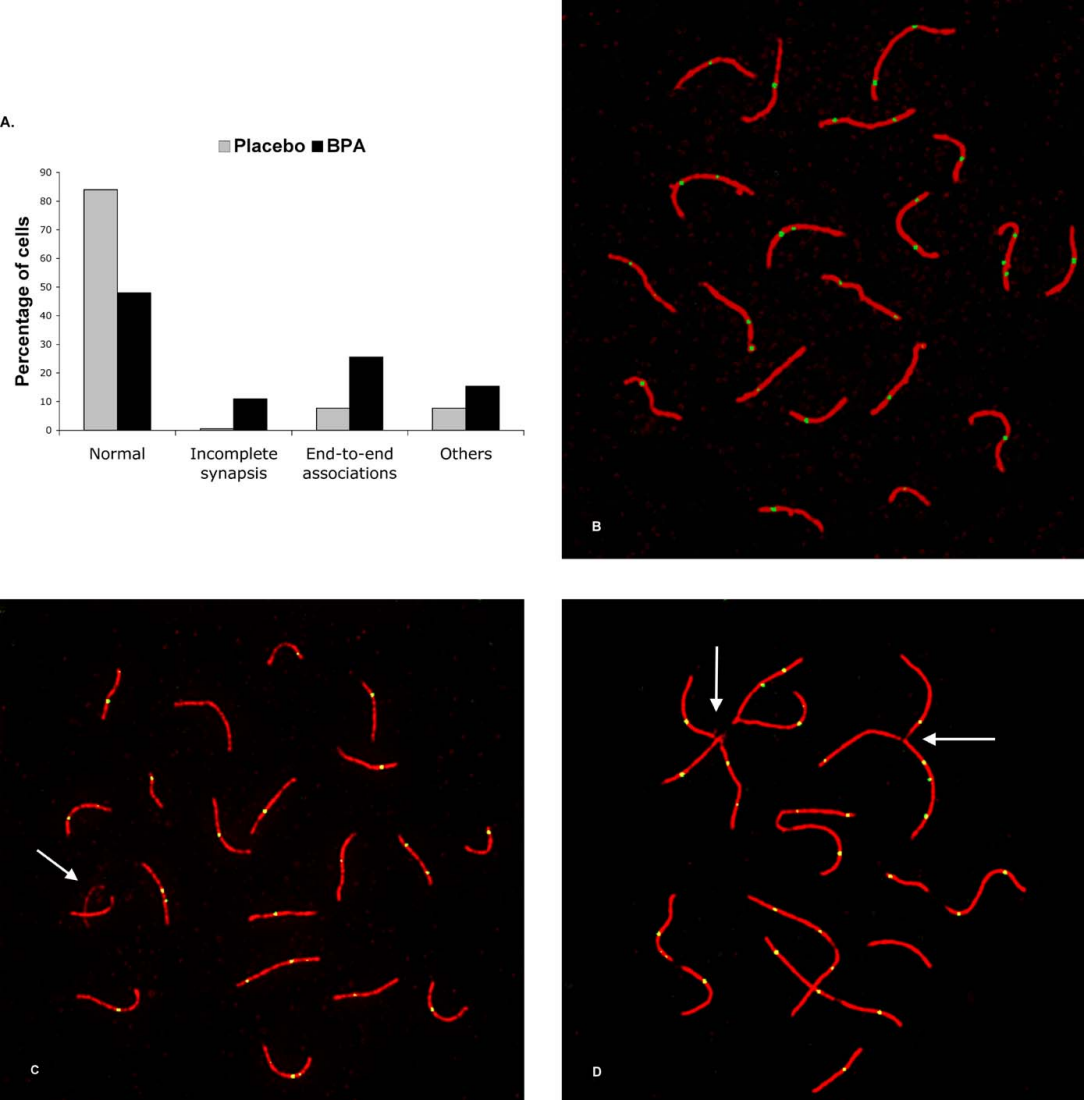
Pachytene Analysis (A) Frequency of synaptic abnormalities in 402 pachytene cells from ten placebo mice and 648 cells from ten BPA-exposed females. (B) Pachytene oocyte from placebo-exposed female immunolabeled with SCP3 (red) and MLH1 (green) and showing normal synapsis. (C and D) Pachytene oocytes from BPA-exposed females showing incomplete synapsis of a single pair of chromosomes (arrow) (C) and abnormal end-to-end associations involving multiple SCs (arrows) (D).

We focused subsequent analyses on pachytene oocytes, the stage at which synapsis between homologous chromosomes is complete and the sites of exchange between homologs become detectable as MLH1-positive foci (reviewed in [15]). Using SCP3 and MLH1 antibodies to visualize the synaptonemal complex (SC) and detect exchanges, respectively [15], we analyzed pachytene cells from placebo and BPA-exposed female fetuses. We found a highly significant increase in synaptic abnormalities in oocytes from BPA-exposed females (16.0% versus 52.0% of cells in placebo and BPA, respectively; χ^2^ = 134.8; *p* < 0.0001; Figure 1A), largely attributable to increases in two categories of abnormality: “incomplete synapsis,” in which a single chromosome pair remained unsynapsed in an otherwise normal pachytene cell (0.5% in placebo versus 11.0% in BPA; Figure 1C), and cells with end-to-end associations between nonhomologous SCs (7.7% in placebo versus 25.6% in BPA; Figure 1D). Synaptic aberrations, including the partial or complete synaptic failure of a single chromosome pair, have been reported in a number of meiotic mutants (reviewed in [16]). To our knowledge, however, the end-to-end association abnormality has not been described previously. Although associations were observed in the placebo group, they were markedly different, involving fewer SCs and “looser” associations. Indeed, as the analysis progressed, this aberration became diagnostic of BPA exposure, allowing a blinded scorer to correctly identify a significant proportion of cells as “exposed.” The significance of these associations is unclear. Meiotic cells undergo nuclear reorganization as prophase progresses, with telomeres clustering at the onset of prophase in a “bouquet” formation [17]. Thus, the increase in end-to-end associations as a result of BPA exposure may reflect the failure of normal chromosome movements at the onset of prophase.

Pachytene oocytes from exposed females also displayed striking aberrations in recombination, as assessed by the number and distribution of MLH1 foci along the SCs. A total of 124 cells were analyzed from nine placebo-exposed females, with a pooled mean of 26.0 ± 2.3 foci per cell; MLH1 foci counts were significantly elevated in BPA-exposed females, with a mean of 29.0 ± 3.7 foci per cell from the analysis of 155 cells from ten females (*t* = 7.7; *p* < 0.0001). There was no significant interindividual variation in either group.

**Figure 2:**
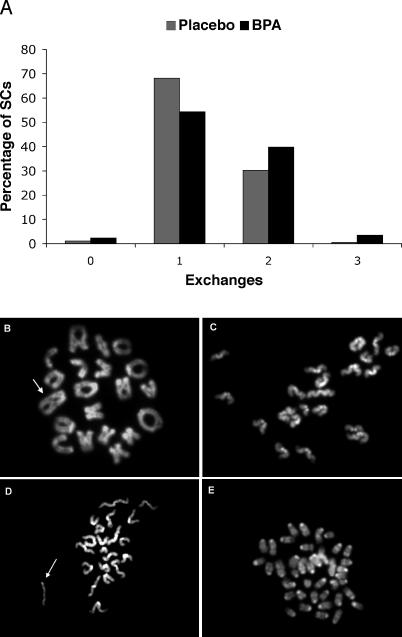
Disturbances in Exchange Frequency and Placement Influence Meiotic Chromosome Segregation (A) Distribution of MLH1 foci. Proportion of SCs with zero, one, two, or three MLH1 foci (exchanges) in pachytene cells from placebo and BPA-exposed females. (B–E) Air-dried chromosome preparations from BPA-exposed females. (B) Diakinesis cell containing a bivalent with three chiasmata (arrow). (C–D) Hyperploid metaphase II eggs with (C) 21 chromosomes and (D) 20 chromosomes plus one prematurely separated sister chromatid (arrow). (E) Hyperploid blastomere with 41 chromosomes from two-cell embryo.

Recombination is regulated by crossover interference, a mechanism that ensures at least one exchange per chromosome pair and controls the proximity of multiple exchanges on a given chromosome (reviewed in [18]). To analyze the distribution of exchanges, we compared the frequency of chromosomes with zero, one, two, or three MLH1 foci (E0, E1, E2, and E3, respectively) in oocytes from placebo and BPA-exposed females (Table 1; Figure 2A). For both groups, the proportion of E0, E1, E2, and E3 bivalents differed significantly from a Poisson distribution (χ^2^ = 1727.9 and χ^2^ = 1568.8 in placebo and BPA, respectively; *p* < 0.0001 in each group), consistent with strong, positive interference. However, we found a modest but significantly altered distribution in exposed females, with an increase in E0s, E2s, and E3s, and a corresponding drop in E1s (χ^2^ = 147.7; *p* < 0.0001; Table 1), suggesting that BPA exposure disrupts the regulation of exchange placement.

**Table 1:**
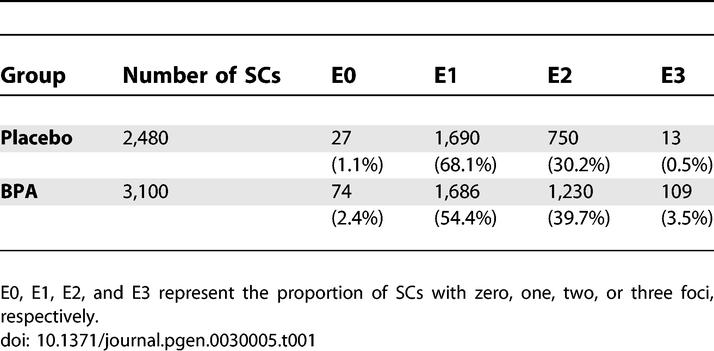
Distribution of MLH1 Foci in Pachytene Cells from Placebo and BPA-Exposed Females

### Recombination Aberrations Verified by Metaphase I Analysis

Previous studies have demonstrated that MLH1 foci at pachytene accurately reflect the sites of exchange [15]. However, the effect of BPA exposure on recombination was unexpected. To verify this observation, we examined recombination using an alternative approach: At metaphase I, both the number and placement of chiasmata can be scored (Figure 2B) and homologous chromosomes that have failed to recombine are easily identified as unpaired univalents. Based on the results of pachytene studies, we predicted that BPA-exposed females would exhibit both an increase in the average number of chiasmata per cell and in univalents. To assess this, three pregnant females implanted with placebo and five implanted with BPA pellets were allowed to go to term, and their offspring were fostered at birth to untreated, lactating females. Oocytes analyzed at 4 wk of age from these in utero–exposed females revealed significant increases in both the average number of chiasmata per cell (25.2 ± 2.5 in placebo versus 27.0 ± 3.1 in BPA; *t* = 3.1; *p* < 0.01; Table 2) and in the frequency of univalents (0.0% in placebo versus 4.4% in BPA; χ^2^ = 4.6; *p* < 0.05; Table 2). Further, the frequency of bivalents with three chiasmata was also increased, although not significantly (Table 3). None of the values precisely matched the pachytene data (e.g., compare Tables 1 and 3); however, this likely reflects the difficulty of accurately counting chiasmata, especially when exchanges are closely placed [19]. Nevertheless, both approaches (i.e., MLH1 and chiasmata counts) provide evidence that BPA exposure elevates recombination frequency and disturbs exchange distribution.

**Table 2:**
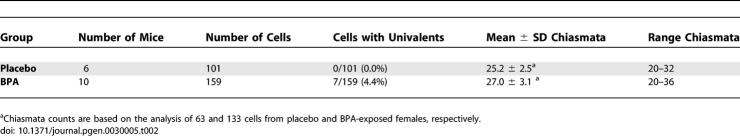
Analysis of Metaphase I Oocytes from Placebo and BPA-Exposed Mice

**Table 3:**
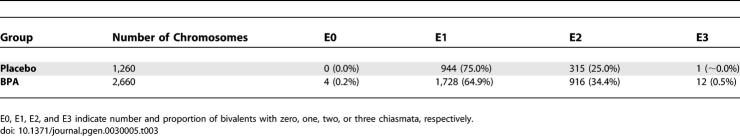
Analysis of Chiasmata Distribution at Metaphase I in Placebo and BPA-Exposed Females

### Increased Aneuploidy in Eggs and Embryos from Adult Females

In humans, aberrations in recombination are associated with meiotic nondisjunction. Both differences in the number of exchanges and their placement along the length of the chromosome (i.e., too close to the centromere or too close to the telomere) have been reported to play a role in the genesis of human trisomy (reviewed in [20]). On this basis, we predicted that the meiotic defects induced by fetal BPA exposure would act to increase aneuploidy in eggs and embryos from adult females.

To assess meiotic nondisjunction, ten pregnant females implanted with placebo and 16 implanted with BPA pellets were allowed to go to term and their offspring (17 placebo and 24 BPA-exposed females) were fostered at birth as described above. At 4–5 wk of age, these females were used either as oocyte or embryo donors for analysis of air-dried chromosome preparations from metaphase II–arrested eggs and two-cell embryos, respectively.

Because analysis of eggs is limited to a single cell, aneuploidy levels are usually estimated by doubling the frequency of hyperploidy to avoid artifacts introduced by chromosome loss. Typical aneuploidy levels for eggs in the laboratory mouse are between 0.5% and 1.0% [21]. We found a significant increase in the level of hyperploid eggs in the BPA group: 1.8% of cells had more than the expected 20 chromosomes in the placebo group compared to 21.4% in the BPA group (χ^2^ = 11.0; *p* < 0.001; Table 4; Figure 2C and 2D). Assuming that hyperploidy represents one half of all nondisjunction, our data suggest that as many as 40% of eggs from females exposed to BPA in utero may be chromosomally abnormal.

**Table 4:**
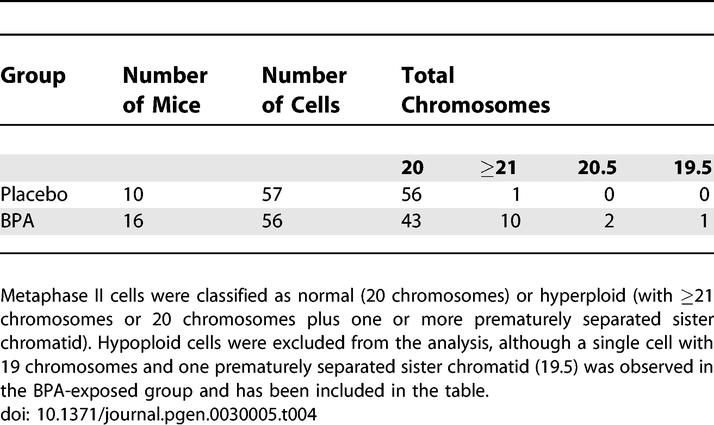
Aneuploidy Analysis

To assess aneuploidy in embryos from exposed females, we superovulated 4- to 5-wk-old females, mated them with wild-type males, and analyzed two-cell embryos. The level of hyperploidy in embryos closely matched the level in eggs (0/13 or 0.0% in placebo versus 4/19 or 21.1% in BPA-exposed; Figure 2E) but the difference between groups was not significant due to the small sample size.

### Studies of Estrogen Receptor Knockout Mice

BPA is considered a “weak” estrogen due to its low binding affinity for the known estrogen receptors; however, its ability to act as a highly potent estrogen mimic at very low concentrations has been demonstrated (reviewed in [22]). Further, in some cases, BPA-induced responses can be mediated through nongenomic mechanisms [23]. To determine whether BPA exerts its effect on the prophase oocyte via a classical estrogen receptor–mediated mechanism, we utilized mice with targeted disruptions of the two known receptors, αERKO and βERKO [24]. Our assumption was that, if BPA acts through one of these receptors, absence of the receptor would make null females insensitive to BPA.

**Figure 3:**
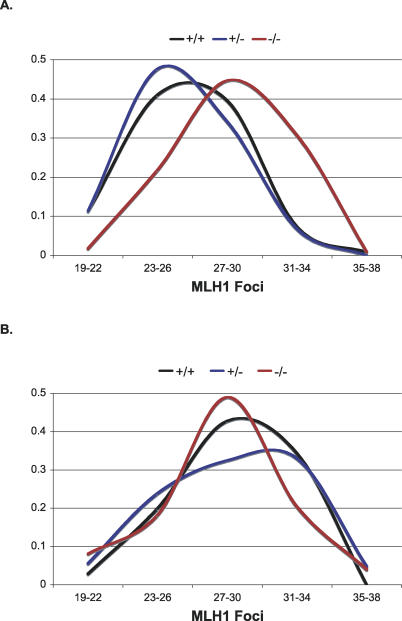
Analysis of Exchanges in Pachytene Oocytes from Unexposed and BPA-Exposed βERKO Females (A) For unexposed animals, there was no difference in mean number of MLH1 foci/cell between wild-type (26.3 ± 3.0) and heterozygous (25.8 ± 2.8) females, but unexposed mutants (28.7 ± 3.2) were highly significantly increased over wild type (*t* = 6.0; *p* < 0.001). These data represent the results from five unexposed pregnant females. For +/+ animals, 124 cells were analyzed from four females; for +/−, 44 cells from three females; and for −/−, 114 cells from four females. Data are provided as mean ± standard deviation. (B) Among exposed animals, the mean values for the three genotypes were virtually identical, but all had highly significantly elevated means over that of unexposed wild-type animals (28.6 ± 3.5, *t* = 3.5, *p* < 0.001; 28.1 ± 3.5, *t* = 4.5, *p* < 0.001; 28.1 ± 3.5, *t* = 3.9, *p* < 0.001 for +/+, +/−, and −/−, respectively). These data represent the results from six pregnant females implanted with BPA pellets. For +/+ animals, 35 cells were analyzed from two females; for +/−, 136 cells from six females; and for −/−, 89 cells from five females. Data are provided as mean ± standard deviation.

Although the data from αERKO females paralleled that of wild-type females (unpublished data), βERKO mice yielded a surprising meiotic phenotype that did not fit our expectation: Pachytene oocytes from unexposed ERβ −/− females exhibited virtually identical defects to BPA-exposed wild-type females. Specifically, we observed similar levels of synaptic aberrations (57.0% of pachytene cells from unexposed ERβ −/− females, as compared to the 52.0% level in BPA-exposed wild-type females in Figure 1A) and increased levels of recombination (Figure 3A). Further, BPA exposure elicited no additional effect in the mutant (Figure 3B). Two important conclusions derive from these findings. First, the meiotic phenotype of the βERKO female implies that ERβ (and, hence, estrogen) plays an important role in the prophase events necessary for recombination during female meiosis. Although sex-specific differences in recombination rate are well documented [15], little is known about the control of recombination in mammals, and, to our knowledge, estrogen has never been implicated. Thus, our findings have important ramifications for the study of recombination. Second, the finding that BPA exposure mimics the effects of an ERβ loss-of-function mutation suggests that, in this system, BPA acts as an estrogen antagonist, not an estrogen mimic.

## Discussion

Previous studies in our laboratory of female mice exposed as young adults [12] provided evidence that low-dose (20 μg/kg/day) short-term BPA exposure during the final stages of oocyte growth increases the likelihood of producing an aneuploid gamete. Consistent with this observation, in vitro studies of BPA exposures in both mitotically dividing somatic cells [25–27] and oocytes undergoing the first meiotic division [13] indicate that BPA adversely affects spindle formation, centrosome dynamics, and chromosome alignment and segregation. Thus, it seems likely that BPA-related abnormalities resulting from in vivo exposure during the late stages of oocyte growth reflect similar effects on the cell division machinery.

In contrast, the studies described herein reveal an effect of BPA on meiotic chromosome segregation by a second, and completely independent, mechanism, that is, by disturbing synapsis and recombination between homologs in the fetal ovary. The finding that unexposed ERβ-null females exhibit a similar phenotype—and that the phenotype cannot be enhanced by BPA exposure—suggests that BPA exerts its effects on the fetal ovary by interfering with ERβ-mediated cellular responses. Knockouts of key meiotic genes involved either in synapsis (e.g., [28,29]) or in the repair of double-strand breaks as recombination events (e.g., [30]) also have demonstrated synaptic and recombination defects. The disturbances both in female fetuses exposed to BPA and in ERβ-null females are notable, however, for two reasons: first, the end-to-end association between the centromeric ends of nonhomologous chromosomes that we observed in a significant proportion of pachytene cells suggests a fundamental disturbance in the early events of chromosome pairing and alignment. To our knowledge, this type of defect has not been reported previously and further studies of early prophase stages to understand this phenotype are in progress. Second, and equally novel, is the finding that, despite disturbances in synapsis, recombination levels are increased in oocytes from exposed females and in females lacking ERβ. This is in marked contrast to other meiotic-disturbance scenarios—not only in mice, but also in lower eukaryotes—in which disturbances in synapsis are accompanied by a decrease in recombination levels (e.g., [28,31,32]). Because little is known about the control of recombination number and placement in mammals, BPA-exposed and ERβ-null females provide a powerful tool, and expression studies to evaluate the gene changes that accompany the observed increase in recombination are currently ongoing.

Defects in synapsis and altered levels of recombination have been correlated with increased aneuploidy in a variety of eukaryotic species. In addition, in humans, subtle changes in the placement of exchanges are correlated with meiotic nondisjunction; indeed, aberrant recombination is the only known molecular correlate of meiotic aneuploidy (reviewed in [20]). To determine whether the altered synaptic and/or recombination patterns in BPA-exposed females increased the likelihood of segregation errors during the first meiotic division, we analyzed females exposed in utero and fostered at birth to untreated mothers. Consistent with studies in both humans and mice (reviewed in [20]), the altered synaptic and recombination profiles we observed at the onset of female meiosis were correlated with increased aneuploidy in eggs and embryos from mature females. Thus, our results provide evidence for a multigenerational effect on chromosome segregation, since daughters of treated pregnant females have an increased risk of producing aneuploid offspring.

While these are worrying possibilities, the implications of our findings are actually much broader: synaptic and recombination defects typically result in the loss of a significant proportion of oocytes prior to sexual maturation [33], reducing the pool of oocytes in the adult female. Thus, in addition to reducing the genetic quality of their eggs and embryos, BPA may adversely influence the reproductive lifespan of exposed females. Experiments to test this prediction are currently ongoing.

Further, because oocytes in the fetal ovary are not yet enclosed in primordial follicles, our findings raise the intriguing possibility that, during the earliest stages of oogenesis, the oocyte is directly responsive to estrogen and to chemicals that can bind ERβ. Although localization studies of ERβ in the adult mouse ovary demonstrate the presence of the receptor largely in granulosa cells [34], previous studies of fetal and adult oocytes in human, bovine, and hamster [35–37] and of spermatocytes in adult rodents [38,39] suggest that ERβ is expressed in premeiotic germ cells and in prophase gonia. These localization studies, coupled with our data demonstrating significant meiotic disturbances in fetal oocytes from ERβ females and in females exposed to BPA, provide compelling evidence that estrogen plays a role in mouse oogenesis far earlier than previously suspected.

An obvious and important question is whether the effects observed in mice can be translated to humans. BPA levels in the parts per billion range have been reported in human serum and amniotic fluid [4–6], and an association between serum BPA levels and recurrent miscarriages in humans has been suggested [40]. Assessing human risk is difficult, and although our data do not allow us to draw conclusions about BPA effects in humans, they demonstrate that a chemical whose actions influence early germ cell development has the potential to induce a three-generation effect when the exposure occurs during pregnancy. Clearly understanding the basis of this effect and, more generally, the influence of estrogen on the early stages of oocyte development are essential first steps in evaluating the potential risk of in utero exposure to chemicals that mimic the action of this hormone.

## Materials and Methods

### Mouse information.

All wild-type mice used in the study were on the C57BL/6 inbred strain background. They were housed in ventilated rack caging in a pathogen-free facility, with drinking water provided in glass water bottles and mouse chow (Purina 5010, http://www.purina.com) provided ad libitum. αERKO and βERKO mice were created by Ken Korach and generated for these studies from heterozygous breeding pairs obtained from Taconic (http://www.taconic.com). Offspring were genotyped by PCR analysis of genomic DNA from ear punch or tail snip tissue using primer sequences provided by Taconic. All animal experiments were approved by the Institutional Animal Care and Use Committee of Case Western Reserve University or Washington State University. Both institutions are fully accredited by the American Association for Accreditation of Laboratory Animal Care.

### Exposure information.

For exposures, BPA or placebo pellets (Innovative Research of America, www.innovrsrch.com) were implanted according to manufacturer guidelines in pregnant females at 11.5 d of gestation. Pellets were designed to release 400 ng of BPA daily, with doses calculated assuming an average weight of 20 g for sexually matured females. This dose was chosen based on our previous studies of BPA exposure in young adult females [12]. For prophase, metaphase I, and metaphase II analyses, exposure experiments were replicated a minimum of three times. Within litters, only female pups of similar developmental stage/weight were included in the analysis (e.g., developmentally delayed or growth-retarded females were excluded). There was no obvious difference in litter size between BPA- and placebo-treated mothers.

### Isolation, culture, and analysis of oocytes/embryos.

To obtain prophase oocytes, pregnant females were killed at 18.5 d of gestation and fetal ovarian tissues prepared as described previously [14]. For analysis of metaphase I, metaphase II, or early cleavage divisions, female offspring were delivered at term, fostered to untreated, lactating females, and matured to 4–5 wk of age. For analysis of metaphase I and metaphase II, germinal vesicle-stage oocytes were retrieved and cultured for 1–2 or 16 h, respectively, as described previously [41]. For analysis of early cleavage stages, embryos were retrieved from oviducts of superovulated females [42]. All chromosome preparations were made using a modification of the Tarkowski technique [43] and scoring was done by two independent observers who were blinded with respect to the status (placebo or BPA-exposed) of the specimen.

### Pachytene analysis.

Synaptonemal complex preparations were made [14] and immunostained with antibodies to SCP3 and MLH1 to analyze synapsis and recombination, respectively, as described previously [44,45]. Analysis of meiotic prophase was conducted in three steps. To assess meiotic progression, 100 meiotic cells were selected at random and substaged as leptotene, zygotene, pachytene, or diplotene on the basis of SCP3 staining. In the second phase of the analysis, cells staged as being at the pachytene stage were scored for synaptic defects. The pachytene-stage cells were grouped into four categories on the basis of synaptic phenotype: 1) normal, if all 20 bivalents exhibited complete synapsis; 2) incomplete synapsis, if one or two pairs of homologs remained unsynapsed in an cell that otherwise exhibited complete synapsis; 3) end-to-end associations, if two or more bivalents exhibited an end-to-end association with no greater than the width of an SC separating them; and 4) other minor synaptic defects such as gaps or fragmentation of the SC. In the final stage of the analysis, MLH1 foci were scored in the subset of pachytene cells exhibiting normal synapsis; this aspect of scoring was conducted by two independent observers who were blinded with respect to the status (placebo versus BPA-exposed and wild type versus mutant).

### Statistical analysis.

Statistical evaluations of possible between-group differences in the mean numbers of MLH1 foci or chiasmata were carried out using standard *t*-test analyses. Goodness-of-fit analyses were used to assess possible between-group differences in the proportion of synaptic defects, distribution of bivalents with zero to three MLH1 foci, proportion of univalents at metaphase I, and proportion of hyperploidy at metaphase II. In any instance in which multiple comparisons were made (e.g., see Figure 3), we adjusted the significance level by applying the Bonferroni correction.

## Abbreviations

BPA: bisphenol A
DES: diethylstilbestrol
SC: synaptonemal complex
